# Surveying of acid-tolerant thermophilic lignocellulolytic fungi in Vietnam reveals surprisingly high genetic diversity

**DOI:** 10.1038/s41598-019-40213-5

**Published:** 2019-03-06

**Authors:** Vu Nguyen Thanh, Nguyen Thanh Thuy, Han Thi Thu Huong, Dinh Duc Hien, Dinh Thi My Hang, Dang Thi Kim Anh, Silvia Hüttner, Johan Larsbrink, Lisbeth Olsson

**Affiliations:** 1 Center for Industrial Microbiology, Food Industries Research Institute, Thanh Xuan, Hanoi Vietnam; 2 Wallenberg Wood Science Center, Department of Biology and Biological Engineering, Division of Industrial Biotechnology, Chalmers University of Technology, SE-412 96 Gothenburg, Sweden

## Abstract

Thermophilic fungi can represent a rich source of industrially relevant enzymes. Here, 105 fungal strains capable of growing at 50 °C and pH 2.0 were isolated from compost and decaying plant matter. Maximum growth temperatures of the strains were in the range 50 °C to 60 °C. Sequencing of the internal transcribed spacer (ITS) regions indicated that 78 fungi belonged to 12 species of Ascomycota and 3 species of Zygomycota, while no fungus of Basidiomycota was detected. The remaining 27 strains could not be reliably assigned to any known species. Phylogenetically, they belonged to the genus *Thielavia*, but they represented 23 highly divergent genetic groups different from each other and from the closest known species by 12 to 152 nucleotides in the ITS region. Fungal secretomes of all 105 strains produced during growth on untreated rice straw were studied for lignocellulolytic activity at different pH and temperatures. The endoglucanase and xylanase activities differed substantially between the different species and strains, but in general, the enzymes produced by the novel *Thielavia* spp. strains exhibited both higher thermal stability and tolerance to acidic conditions. The study highlights the vast potential of an untapped diversity of thermophilic fungi in the tropics.

## Introduction

Thermotolerance is not very common among eukaryotes. While the upper temperature limit for the growth of prokaryotes has been reported to be 121 °C^1^, the highest growth temperature of eukaryotes is around 60–62 °C^2^, and only a small number of fungal species thrive at such high temperatures. Among the estimated 3.0 million fungal species existing in nature, and approximately 100 000 species described, only about 50 species have been found to be able to grow at 50–60 °C^3^. These species are limited to the Sordariales, Eurotiales, and Onygenales in the Ascomycota and the Mucorales of the Zygomycota. No representative of thermophilic Basidiomycota has yet been confirmed^4^. Fungi capable of growing at elevated temperatures are classified into thermophilic and thermotolerant groups. There is no consensus on the demarcation between the two groups, but typically, a fungus that has a thermal maximum near 50 °C and a minimum below 20 °C is regarded as thermotolerant, while those that grow at 50 °C or above, but not grow at 20 °C or above^5^ are regarded as thermophilic. In the present study, the cultivation temperature was maintained at 50 °C during initial screening for thermophilic fungi.

Earlier reports of thermophilic fungi were the result of accidental contamination of organic materials incubated at elevated temperatures. These include the isolation of *Mucor pusillus* (=*Rhizomucor pusillus*) from bread in 1886, and *Humicola lanuginosa* (=*Thermomyces lanuginosus*) from potato slices in 1899 (from Johri *et al*.^6^). It was later found that thermophilic fungi are a regular microbial component of self-heating decomposing hay^7^. In natural environments, thermophilic fungi are most commonly found in rapidly decomposing plant residues, where heat is generated through exothermic microbial activity. Heat accumulation in a 5-cm layer of leaf litter is sufficient to create favourable conditions for thermophilic fungi, and temperature increase may even lead to ignition in stockpiles of hay, oil seeds or manure. Most thermophilic fungi also grow well at moderate temperatures and can be found in various substrates, including soils, composts, piles of hay, stored grains, wood chip piles, nesting materials of birds and animals or in municipal refuse^8^.

While thermotolerant/thermophilic fungi appear to be exceedingly rare, fungi able to tolerate acidic conditions are frequently encountered in nature, and many species have been shown to be capable of growing at pH levels as low as 2^9^. There is no clear demarcation between acidophilic and acid-tolerant fungi, but it is often assumed that acidophilic fungi are those that can grow at pH 1.0 and have optimum growth at pH 3.0 or below^10^. Hereafter, we refer to acidophilic species as those optimally growing at pH values below 3, while acidotolerant species refer to species able to grow in acidic environments but with growth optima above pH 3. The earliest description of acidophilic fungi dates back to 1943, when a strain of *Acontium velatum* and a “Fungus D” were shown to be capable of growing in glucose medium containing 1.25 M sulphuric acid at pH 0^11^. Unfortunately, the strain of *Acontium velatum* appears to have been lost since the initial publication, but “Fungus D” is now believed to be a strain of *Acidomyces acidophilus* which is commonly found in extremely acidic environments^12^. Until now, acidophility has been shown for only 6 fungal species, including *Acidomyces acidophilus* (MB#511856) (=*Scytalidium acidophilum* = *Acidomyces richmondensis* = Fungus D), *Acidomyces acidothermus* (MB#564520), *Acidothrix acidophila* (MB#805424), *Acidea extrema* (MB#805425), *Acontium velatum* (MB#142596, no living specimen available) and *Hortaea acidophila* (MB#367373) (=*Neohortaea acidophila*). The strain *Bispora* sp. MEY-1, well-known for the production of a range of thermophilic and acid-tolerant lignocellulolytic enzymes^13^, probably belongs to the species *Acidomyces acidothermus*^14^. Phylogenetically, all acidophilic species are Ascomycota, and the teleomorphic state is known only for *Acidomyces acidothermus* (described as *Teratosphaeria acidotherma*, MB#517415)^14^.

Thermophilic and acid-tolerant fungi have received considerable attention, as their thermostable enzymes can be employed in industrial processes at elevated temperatures. Increasing the process temperature can have advantages, for example, increasing the rate of chemical reactions, decreasing the viscosity of substrates and reducing the risk of contamination by mesophilic microorganisms^15^. A number of enzymes such as amylases, cellulases, xylanases, lipases and proteases from thermophilic fungi have been found to be thermostable^6^. The lipase and rennet from *Rhizomucor miehei* are commercial enzymes that find wide applications in the food industry^16^. A range of genes encoding lignocellulolytic enzymes in the acidophilic fungus *Acidomyces acidothermus* MEY-1 has been cloned and expressed. The enzymes were found to be thermotolerant and functional under acidic conditions^13^.

As a result of increased interest in renewable sources of energy and biomaterials, fungi that are adapted to extreme environmental conditions have recently received special interest as sources of novel hydrolytic enzymes suitable for various technological applications. Genome sequences have been obtained for a large number of thermophilic fungi, such as *Myceliophthora thermophila*, *Thielavia terrestris*, *Thielavia heterothallica*, *Chaetomium thermophilum*, *Thermomyces lanuginosus*, *T. thermophilus*, *Rhizomucor miehei*, *Talaromyces cellulolyticus* and *Malbranchea cinnamomea*, as well as for acidophilic fungi, including *Acidothrix acidophila*, *Acidomyces acidophilus* and *Hortaea acidophila*^17^.

Genomes of thermophilic lignocellulose-degrading fungi such as *Thermothelomyces thermophila*^18^, *Thielavia terrestris*^18^, and *Malbranchea cinnamomea*^19^ have been found to harbour large numbers of carbohydrate-active enzymes (CAZymes). For examples, *Thielavia terrestris* genome encodes 473 CAZymes, including 212 glycoside hydrolases (GHs), 91 glycosyl transferases (GTs), 4 polysaccharide lyases (PLs), 28 carbohydrate esterases (CEs), 58 enzymes with auxiliary activities (AAs) and 80 carbohydrate-binding modules (CBMs). Compared to the well-known cellulase-producer *Trichoderma reesei*, *Thielavia terrestris* has a similar setup of GHs^18^. Through studies of the genomes of thermophilic fungi compared to related mesophiles, it appears that common strategies for thermal adaptation include a reduction of the genome size and an increased frequency of the amino acids Ile, Val, Tyr, Trp, Arg, Glu, and Leu (IVYWREL) in proteins^20^. Although high GC mol% is often assumed to contribute to the genome stability at elevated temperatures, the correlation between GC content and thermophilicity in fungi remains inconclusive^21^.

To take advantage of the high fungal biodiversity in the tropics, we have conducted a search in Vietnam for novel fungi for the production of lignocellulolytic enzymes applicable in agriculture and the bioconversion of plant biomass. In a previous study, we reported on the screening of 1100 mesophilic fungal isolates from decaying plant tissue for cellulase, xylanase and accessory enzyme activities^22^. In the present work, we aimed to explore the biodiversity in northern Vietnam to identify filamentous fungi able to grow at elevated temperatures (50 °C) under extremely acidic conditions (pH 2.0) using untreated rice straw as the sole carbon source. The underlying hypothesis was that the enzymes produced by the isolated fungi would be thermostable and functional under acidic conditions. Such enzymes are highly relevant for the feed industry, especially to improve the nutritional content of animal feed, where the enzymes must be stable at high temperatures during the pelleting process and also be functional in the acidic conditions of the animal’s stomach.

## Results and Discussion

### Fungal diversity

After 7–10 days of incubation of pre-washed plant debris on medium containing rice straw as sole carbon source at 50 °C, the proliferation of fungal hyphae was observed at pH 2.0 and 3.0, but not at pH 1.0. Moderate selection power was achieved at pH 2.0 and typically each plate contained one or rarely two macroscopically (colour and texture) distinctive types of fungi. This pH was employed for the whole study. A total of 105 fungal strains were obtained from 78 samples of compost and various kinds of decaying plant matter. The list of isolates and isolation source are presented in the Supplementary Dataset S1.

Although the fungi were isolated at pH 2.0, when inoculated in liquid media with different pHs they did not grow at pH 2.0 and showed only weak growth at pH 2.5, whereas growth at pH 3.0 and 5.0 was strong. Therefore, all the isolates were regarded as acidotolerant. We assume that, during initial isolation, the pH inside the inoculants (plant residues) might have been higher than that in the medium due to mechanical barrier or pH buffer capacity of the inoculants.

**Figure 1:**
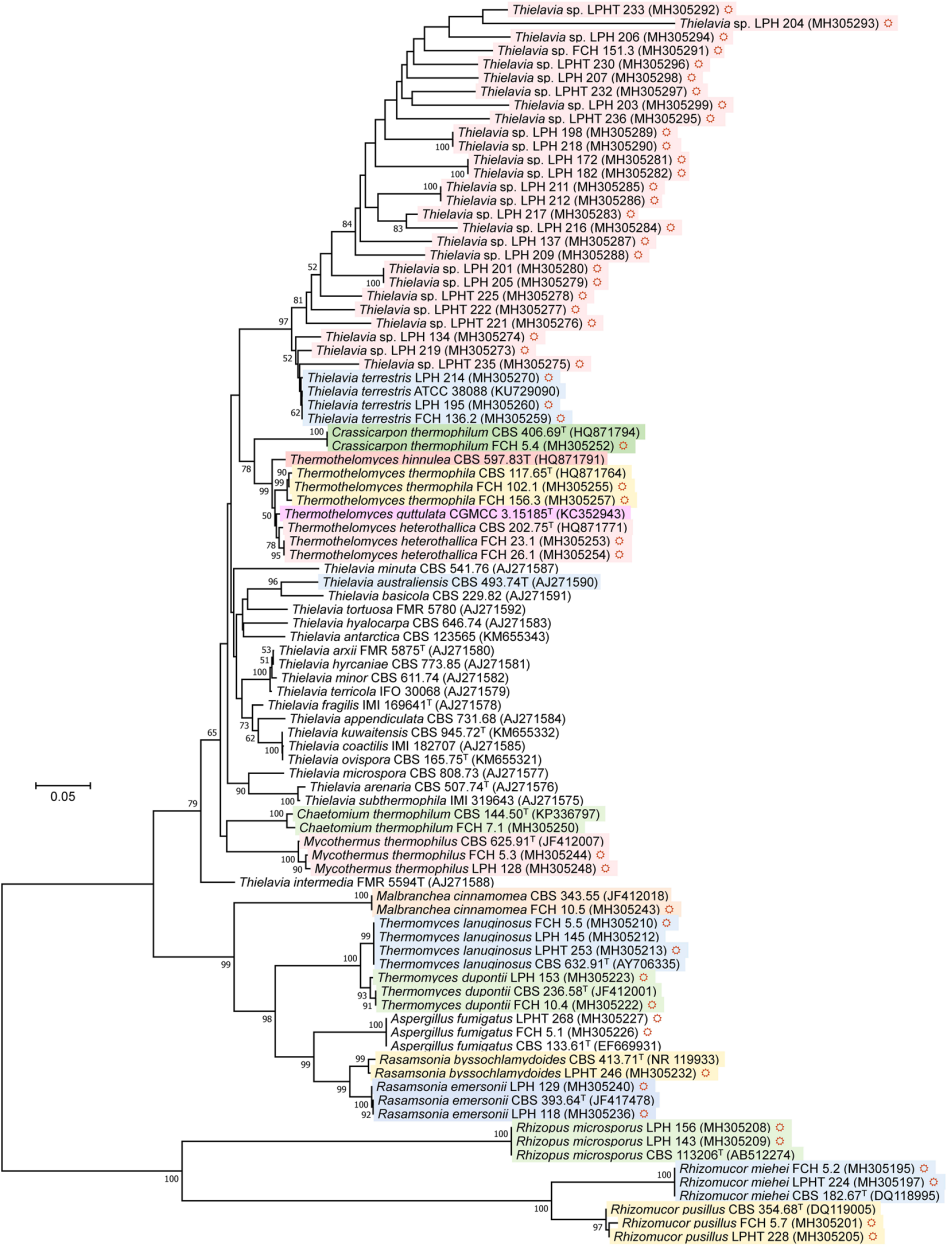
Neighbour-joining phylogenetic tree showing the extreme genetic divergence of *Thielavia* strains isolated from Vietnam, and their relationship to known *Thielavia* and thermophilic species. The tree was constructed based on ITS sequences using the maximum composite likelihood method in MEGA7. An alignment of 623 positions corresponding to 439 nucleotides for *Thermomyces lanuginosus* CBS 632.91^T^ was taken into analysis. All ambiguous positions were removed for each sequence pair. Bootstrap values of > 50%, obtained from 1000 replications, are shown. GenBank accession numbers of ITS sequences are given in parentheses. Bar, 5% sequence divergence; strains obtained in this study; shaded, thermophilic taxa.

On the basis of the ITS sequence similarity to the published type/reference strains, 78 strains were closely affiliated with 15 known taxa, while the remaining 27 strains could not be reliably identified. Most of the isolates belonged to Ascomycota (89 strains), and the remaining belonged to Zygomycota (16 strains). Regarding species richness among these genera, the diversity of *Thielavia* was striking. As will be discussed below, among 42 isolated strains, 24 genetic groups of *Thielavia* that differed from each other at the species level were detected (Fig. 1). The less diverse genera were those that have been reported to occur commonly in compost, such as *Thermomyces*, *Rhizomucor*, *Mycothermus* and *Aspergillus*^21^. A relatively high number of isolates was found for one or two species within these genera. In term of frequency, the species could be listed in following decreasing order: *Thielavia terrestris* (15 strains), *Thermomyces lanuginosus* (12 strains), *Rasamsonia emersonii* (9 strains), *Rhizomucor miehei* (7 strains), *Aspergillus fumigatus* (6 strains), *Mycothermus thermophilus* (=*Scytalidium thermophilum*) (6 strains), *Rhizomucor pusillus* (5 strains), *Thermothelomyces thermophila* (=*Myceliophthora thermophila*) (4 strains), *Rhizopus microsporus* (4 strains), *Thermomyces dupontii* (=*Talaromyces thermophilus*) (4 strains), *Thermothelomyces heterothallica* (=*Myceliophthora heterothallica*) (2 strains), *Chaetomium thermophilum* (1 strain), *Malbranchea cinnamomea* (1 strain), *Crassicarpon thermophilum* (=*Myceliophthora fergusii*) (1 strain), and *Rasamsonia byssochlamydoides* (=*Talaromyces byssochlamydoides*) (1 strain) (Table 1). Apart from *Aspergillus fumigatus*, which is known to be thermotolerant^21^, all species found in this study were thermophilic and among the frequent inhabitants of compost and decomposing hay^21^.

**Table 1:** Genetic diversity of thermophilic/thermotolerant fungi isolated from compost, plant residues and soil in Vietnam, assessed from ITS sequences.

Taxon name	MycoBank number	Number of strain	Number of genetic groups	Variation, nuc	Difference from type, nuc/N	Selected strain	GenBank# of selected strain	Type/reference strain	GenBank# of type/reference
Rhizomucor miehei ( = Mucor miehei)	MB#322483	7	2	1	0/6; 1/1	LPHT 224	MH305197	CBS 182.67^T^	DQ118995
Rhizomucor pusillus ( = Mucor pusillus)	MB#322484	5	2	1	0/4; 1/1	FCH 5.7	MH305201	CBS 354.68^T^	DQ119005
Rhizopus microsporus	MB#447066	4	2	1	0/2; 1/2	LPH 156	MH305208	CBS 113206^T^	AB512274
Thermomyces lanuginosus ( = Humicola lanuginosa)	MB#239786	12	1	0	0/12	FCH 5.5	MH305210	CBS 632.91^T^	AY706335
Thermomyces dupontii ( = Talaromyces thermophilus)	MB#805186	4	4	5	0/1; 1/1; 4/1; 5/1	LPHT 269	MH305224	CBS 236.58^T^	JF412001
Aspergillus fumigatus	MB#211776	6	3	3	0/4; 2/1; 3/1	FCH 5.1	MH305226	CBS 133.61^T^	EF669931
Rasamsonia byssochlamydoides ( = Talaromyces byssochlamydoides)	MB#519877	1	1	0	10/1	LPHT 246	MH305232	CBS 413.71^T^	NR_119933
Rasamsonia emersonii ( = Talaromyces emersonii)	MB#519874	9	3	2	0/4; 1/1; 2/4	LPH 118	MH305236	CBS 393.64^T^	JF417478
Malbranchea cinnamomea	MB#106998	1	1	0	1/1	FCH 10.5	MH305243	CBS 343.55	JF412018
Mycothermus thermophilus ( = Scytalidium thermophilum)	MB#807382	6	3	4	2/3; 4/1; 5/1; 6/1	FCH 5.3	MH305244	CBS 625.91^T^	JF412007
Chaetomium thermophilum	MB#427529	1	1	0	8/1	FCH 7.1	MH305250	CBS 144.50^T^	KP336797
Crassicarpon thermophilum ( = Myceliophthora fergusii)	MB#809488	1	1	0	0/1	FCH 5.4	MH305252	CBS 406.69^T^	HQ871794
Thermothelomyces heterothallica ( = Myceliophthora heterothallica)	MB#809491	2	1	0	4/2	FCH 23.1	MH305253	CBS 202.75^T^	HQ871771
Thermothelomyces thermophila ( = Myceliophthora thermophila)	MB#809493	4	3	5	0/1; 3/2; 5/1	FCH 156.3	MH305257	CBS 117.65^T^	HQ871764
Thielavia terrestris	MB#324578	15	4	2	0/4; 1/4; 2/7	FCH 136.2	MH305259	ATCC 38088	KU729090
Thielavia sp. (1)	NA	1	1	0	12/1	LPH 219	MH305273	ATCC 38088	KU729090
Thielavia sp. (2)	NA	1	1	0	12/1	LPH 134	MH305274	ATCC 38088	KU729090
Thielavia sp. (3)	NA	1	1	0	46/1	LPHT 235	MH305275	ATCC 38088	KU729090
Thielavia sp. (4)	NA	1	1	0	41/1	LPHT 221	MH305276	ATCC 38088	KU729090
Thielavia sp. (5)	NA	1	1	0	41/1	LPHT 222	MH305277	ATCC 38088	KU729090
Thielavia sp. (6)	NA	1	1	0	39/1	LPHT 225	MH305278	ATCC 38088	KU729090
Thielavia sp. (7)	NA	2	2	0	41/2	LPH 205	MH305279	ATCC 38088	KU729090
Thielavia sp. (8)	NA	2	2	0	80/2	LPH 172	MH305281	ATCC 38088	KU729090
Thielavia sp. (9)	NA	1	1	0	62/2	LPH 217	MH305283	ATCC 38088	KU729090
Thielavia sp. (10)	NA	1	1	0	70/1	LPH 216	MH305284	ATCC 38088	KU729090
Thielavia sp. (11)	NA	2	2	0	63/1	LPH 211	MH305285	ATCC 38088	KU729090
Thielavia sp. (12)	NA	1	1	0	75/1	LPH 137	MH305287	ATCC 38088	KU729090
Thielavia sp. (13)	NA	1	1	0	65/1	LPH 209	MH305288	ATCC 38088	KU729090
Thielavia sp. (14)	NA	2	2	0	69/1	LPH 198	MH305289	ATCC 38088	KU729090
Thielavia sp. (15)	NA	1	1	0	87/1	FCH 151.3	MH305291	ATCC 38088	KU729090
Thielavia sp. (16)	NA	1	1	0	85/1	LPHT 233	MH305292	ATCC 38088	KU729090
Thielavia sp. (17)	NA	1	1	0	152/1	LPH 204	MH305293	ATCC 38088	KU729090
Thielavia sp. (18)	NA	1	1	0	104/1	LPH 206	MH305294	ATCC 38088	KU729090
Thielavia sp. (19)	NA	1	1	0	94/1	LPHT 236	MH305295	ATCC 38088	KU729090
Thielavia sp. (20)	NA	1	1	0	94/1	LPHT 230	MH305296	ATCC 38088	KU729090
Thielavia sp. (21)	NA	1	1	0	82/1	LPHT 232	MH305297	ATCC 38088	KU729090
Thielavia sp. (22)	NA	1	1	0	96/1	LPH 207	MH305298	ATCC 38088	KU729090
Thielavia sp. (23)	NA	1	1	0	92/1	LPH 203	MH305299	ATCC 38088	KU729090

NA – Not applicable; ^T^ – type strain; nuc/N – number of nuc difference and number of strain (N).

It is interesting to note that the species with the highest occurrence showed little genetic variation between the strains. For example, all 12 *Thermomyces langinosus* strains were identical to the type strain in their ITS regions. Similarly, the species *Thielavia terrestris* (15 strains) and *Rasamsonia emersonii* (9 strains) also showed only 1–2 nucleotide (nuc) (or 0.16–0.32%) internal variation in the ITS sequences. However, high variation was observed in some species with lower occurrence. The 4 strains of *Thermomyces dupontii* were unique, and differed from each other by 1 to 5 nuc (or 0.18–0.91% variation) in their ITS sequences. Similarly, the 6 *Mycothermus thermophilus* strains represent 4 genetic groups with up to 5 nuc (or 0.94%) variations. In total, 59 unique genetic groups were detected within the 105 isolates (Table 1).

The assignment of 78 strains to 15 known species was considered reliable. The studied strains formed well supported clades (bootstrap confidence above 93%) with the type/reference strains of the corresponding taxa (Fig. 1) and nucleotide differences were less than 1%. Exceptions were LPHT 246 and FCH 7.1; the ITS sequence of LPHT 246 was closest to the GenBank sequence NR_119933 of the type strain of *Rasamsonia byssochlamydoides*, but differed from the latter by 10 nuc (1.81% variation). Similarly, the ITS sequence of the strain FCH 7.1 differed from the type strain of *Chaetomium thermophilum* by 8 nuc (1.35% variation). The differences are close to the limit for species delimitation, which is considered to be 1.96% for Ascomycota^23^, and the specific assignments of the strains are therefore provisional. Due to rapid changes, and confusion, in the taxonomy of thermophilic fungi^3^, all species names in this study are listed together with the corresponding MycoBank numbers and synonyms (Table 1).

In this study, 15 isolates were identified as *Thielavia terrestris* based on the ITS sequence similarity (less than 2 nuc difference) to the GenBank sequence KU729090 of the well-studied *Thielavia terrestris* ATCC 38088 strain. The genome of *Thielavia terrestris* ATCC 38088 has been sequenced and analysed^18^. Strictly speaking, the type strain of *Thielavia terrestris* is CBS 355.66 and not ATCC 38088. However, the ITS sequence of CBS 355.66 (CBS Record id: 16190616) differs from ATCC 38088 by only 1 nuc (542/543), and thus, the strains are considered conspecific.

As mentioned above, 27 strains could not be assigned to any known fungal species. Phylogenetically, the strains were found to be related to the genus *Thielavia*, and formed a well-supported clade (97% confidence) with *Thielavia terrestris* (Fig. 1). In the ITS sequences, they were also most close to *Thielavia terrestris* but differed from the latter by 12 to 152 nuc (or 2.16–27.39% variation), and are thus apparently far from being of the same species. Internally, these strains were highly heterogeneous in their ITS sequences, and belonged to 23 genetic groups. Another surprising fact is that each of the new genetic groups was represented by only 1 or 2 strains (19 groups with 1 strain and 4 groups with 2 strains) (Table 1). These 19 groups were nearly independent as indicated by low bootstrap values (Fig. 1). This may imply that the segregation of these genetic groups took place very recently and by some singular events. Otherwise, we might have found more isolates for a certain genetic group or some relatedness among them. The driving force for such rapid segregation is still unclear.

**Figure 2:**
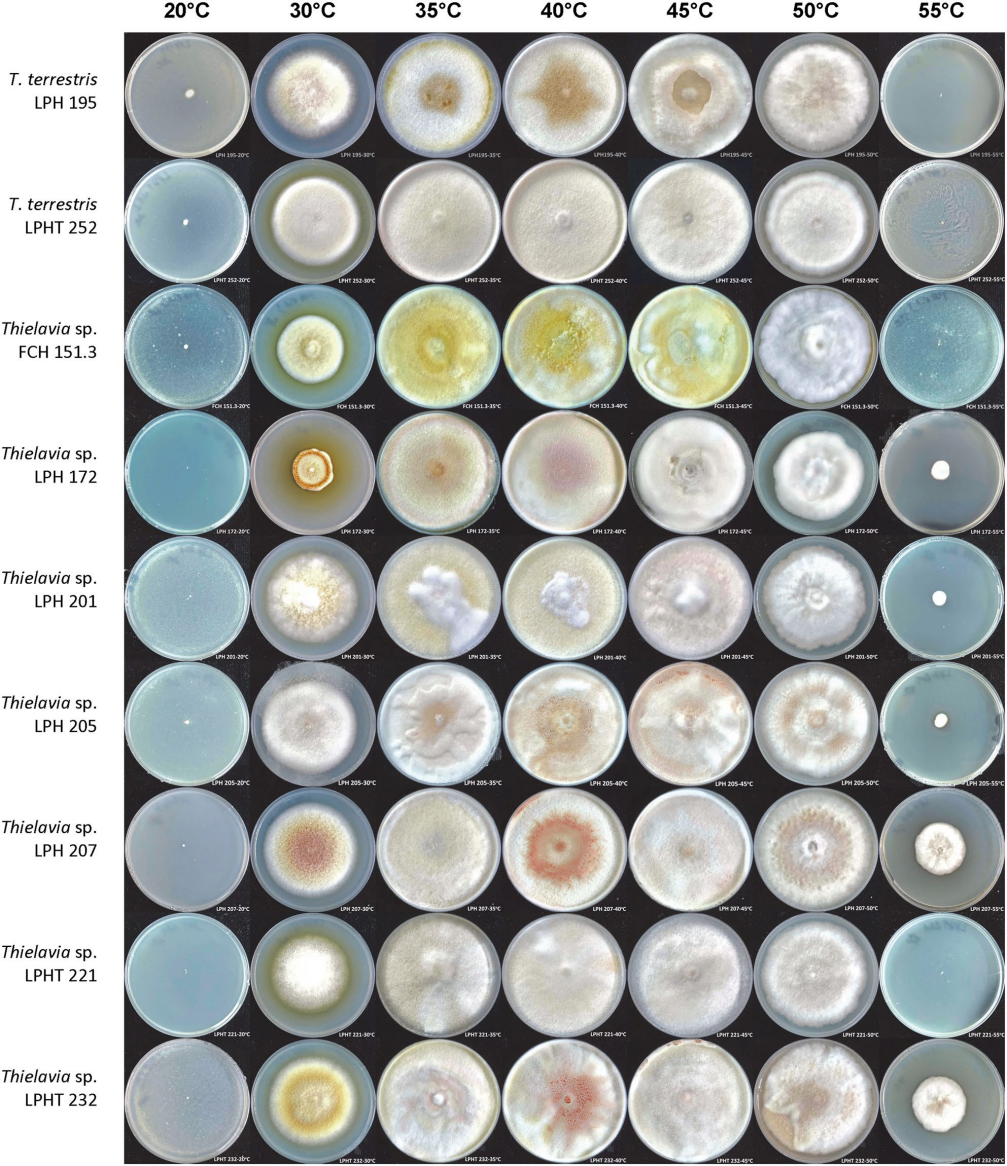
The one-week growth of *Thielavia* strains on PDA agar at different temperatures.

**Figure 3:**
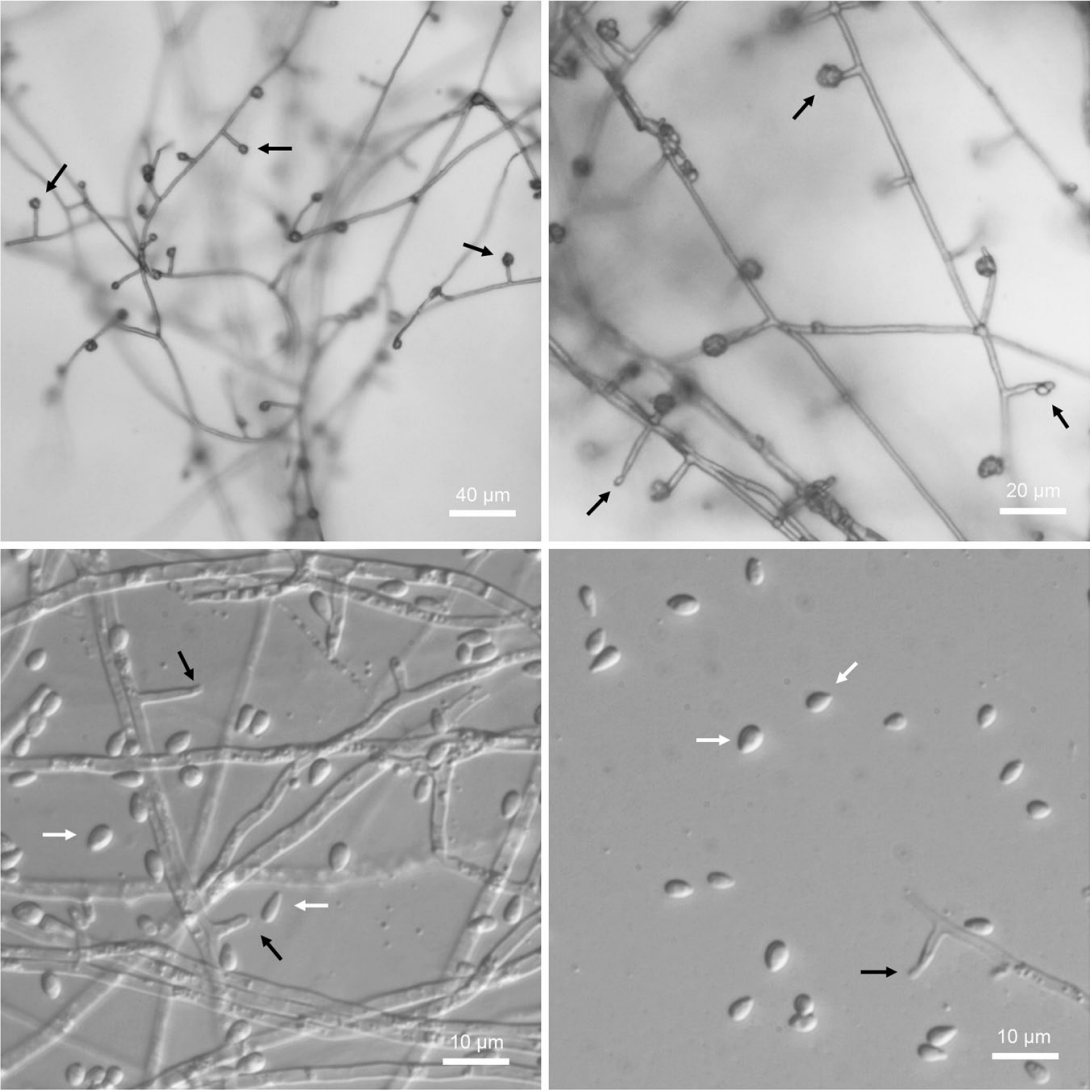
Holoblastic conidiogenesis of *Thielavia* sp. LPH 233 grown on PDA agar at 50 °C. Black arrows, phialides; white arrows, blastoconidia.

The unidentified *Thielavia* strains had optimum growth temperatures in the range 35 °C to 50 °C, and showed no or only limited growth at 20 °C and 60 °C (Fig. 2). Given these thermal growth patterns, these strains have been designated as thermophilic^3^. Bearing in mind the fact that only about 50 species of thermophilic fungi were known previously^3^, the discovery of these 23 new genetic groups is striking. *Thielavia* spp. isolates (both *Thielavia terrestris* and the new genetic groups) were similar in colony appearance (texture, colour) and conidiogenesis. On PDA at 50 °C, *Thielavia* strains formed white cottoneous spreading colonies that gradually changed to pinkish, yellow or light brown. Some strains formed diffused yellow pigment in the medium (Fig. 2). The strains produced rare holoblastic conidia (Fig. 3). No ascomata formation was observed. The genus *Thielavia* (MB#5450) comprises 41 validly described species and among them only 2 are thermophilic, namely *Thielavia australiensis* and *Thielavia terrestris*^21^.

No basidiomycetous fungus was found in the present study. The presence of thermophilic Basidiomycota has long been debated. Two unidentified basidiomycetous isolates have been shown to be able to grow at 45 °C in a study by Straatsma *et al*. ^24^, but no additional studies on growth at different temperatures were conducted, making it impossible to classify the isolates as thermophilic or thermotolerant. The thermophilic species *Myriococcum thermophilum* previously listed as a mitosporic basidiomycete is now classified as *Crassicarpon hotsonii* (MB#816112) under Ascomycota^25^.

Although ITS has been used widely as the primary marker for fungal barcoding^23^, the interpretation of data should be made with caution. ITS might be a poor choice for a certain group of fungi. For example, due to the presence of highly divergent non-orthologous copies of the ITS2 in *Fusarium*, ITS sequences cannot resolve species boundaries in the genus^26^. Protein-coding genes, such as translation elongation factor 1-alpha (tef1), RNA polymerase II largest (RPB1) and second largest subunit (RPB2), provided much better resolution for species of *Fusarium*^26^. Even though ITS was successfully utilised for discrimination of *Thielavia* species^27^, additional markers might be needed for understanding the taxonomic positions of the genetically highly divergent *Thielavia* isolates obtained in this study.

### Lignocellulolytic activity

**Figure 4:**
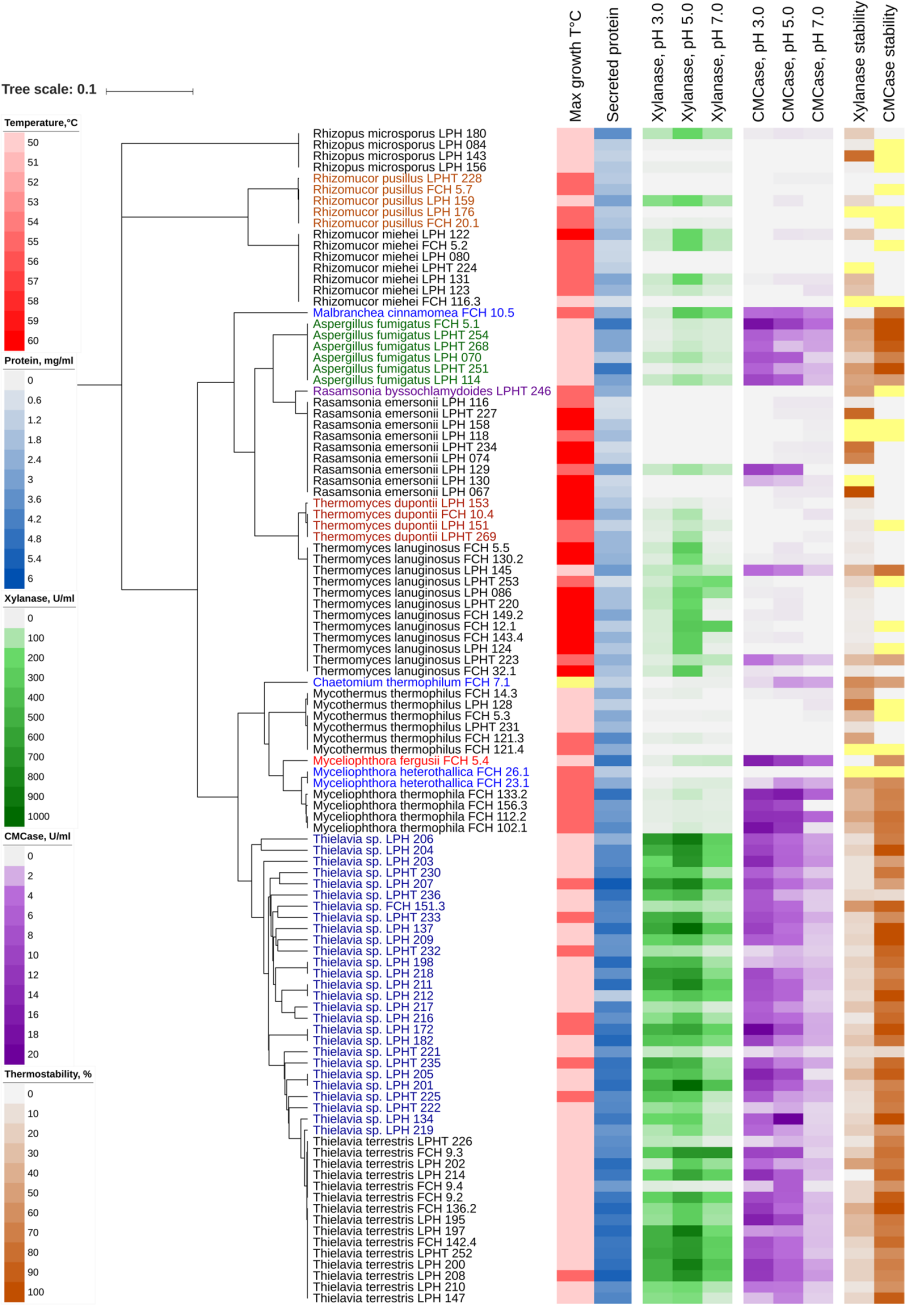
Summary of the properties of the 105 thermophilic/thermotolerant strains obtained in Vietnam. The UPMA phylogenetic tree was constructed in MEGA7 based on ITS sequences using the maximum composite likelihood method. Maximum growth temperatures, secreted protein concentrations, xylanase and CMCase activities of secretomes at different pHs, and residual activities after heat treatment at 70 °C are displayed in heat maps using iTOL. Yellow cells, no data. Numerical data are provided in the Supplementary Dataset S1.

**Figure 5:**
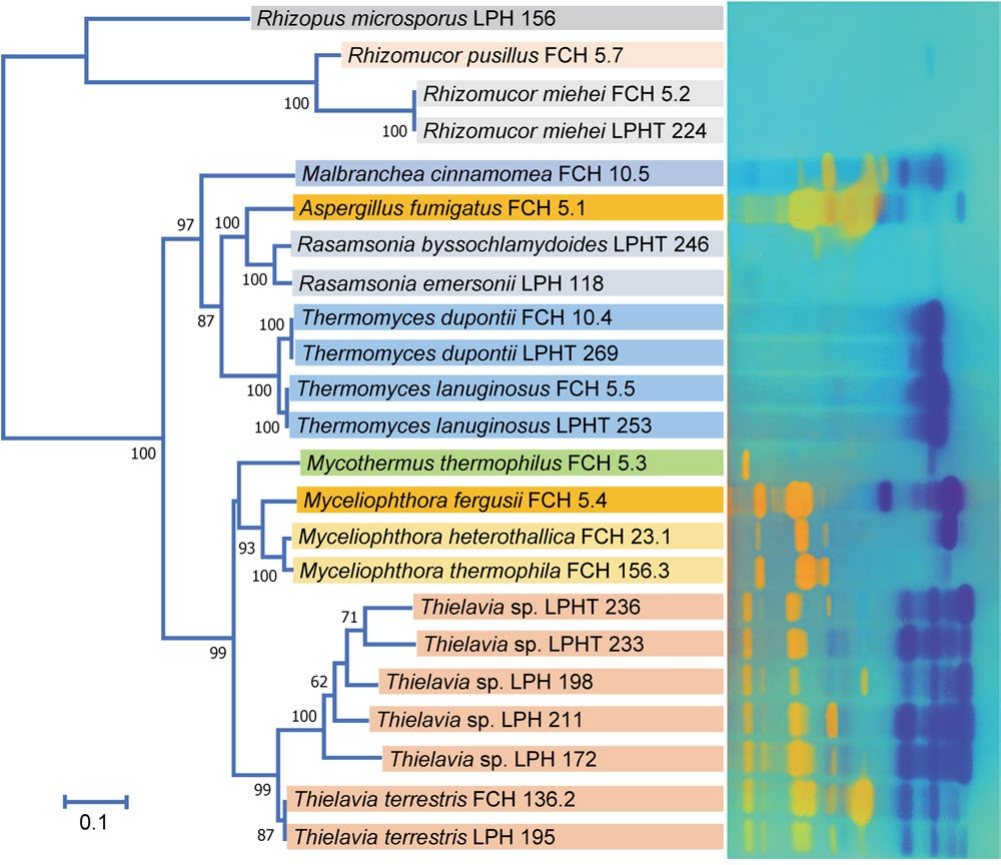
Zymograms of secretomes obtained from selected thermophilic and thermotolerant strains grown on rice straw as the sole carbon source. Photographs of zymogram gels were colorized (CMCase in yellow and xylanase in dark-blue) and overlaid to produce the final image. The original images are provided in the Supplementary S2. The neighbour-joining tree was constructed based on the ITS sequences using MEGA7. Bar, 10% sequence divergence.

An overall significant correlation was found between lignocellulolytic activities and the amount of extracellular protein produced when the isolates were grown on rice straw (Fig. 4). Presumably, the fungi that possess a more effective lignocellulolytic machinery exhibit faster growth and hence excrete more enzymes into the medium. The low lignocellulase activities of several of the fungal species may be explained by the recalcitrance of the untreated rice straw that was used as the cultivation substrate. Zymograms of fungal secretomes were found to correlate well with the liquid enzyme activity assays. Species/strains that demonstrated high enzymatic activities also produced a wider range of enzymes, as indicated by a more varied band pattern on the zymograms (Figs 4 and 5). A combination of phylogenetic and enzymatic analyses indicated that the lignocellulose degradation patterns of closely related species/genera were similar. This could allow the provisional prediction of the ecology and lignocellulose degradation characteristics of a fungus based on its taxonomic position.

The isolated strains belonging to the Zygomycota (*Rhizomucor miehei*, *Rhizomucor pusillus* and *Rhizopus microsporus*) exhibited low xylanase and no carboxymethyl cellulase (CMCase) activities (Fig. 4). These species are not known as strong lignocellulose degraders and their genomes contain only about a half of the number of the CAZymes identified in the lignocellulolytic fungus *Thielavia terrestris*^28,29^. These fast-growing *Mucoraceae* are widespread in nature; they are often the first fungi to occupy moist plant debris^30^ and are known for their ability to degrade starch, proteins and lipids, which are much less recalcitrant substrates compared to lignocellulose. Accordingly, while the genome of *Rhizomucor miehei* harbours only 110 GHs, it encodes 155 proteases and 254 ester hydrolases^29^.

Low lignocellulolytic activities were also observed for the strains of *Rasamsonia emersonii*, *Mycothermus thermophilus*, *Thermomyces lanuginosus* and *Thermomyces dupontii* (Fig. 4). An exception was *R. emersonii* strain LPH 129, which exhibited both xylanase and CMCase activities; the CMCase activity being higher at pH 3.0 than at pH 5.0 or pH 7.0. *Rasamsonia emersonii* is an industrially important fungus that produces enzymes (β-glucanase, cellobiohydrolase, β-glucosidase) used in baking^31^ and beer production^32^. The fungus *Mycothermus thermophilus* is an important fungus in mushroom composting^33^.

Low lignocellulolytic activity of the strains assigned to the genus *Thermomyces* may seem counterintuitive as the genus has been extensively studied in biomass bioenergy research. The relatively low production of xylanase by *Thermomyces* strains when grown on untreated rice straw might be explained by the recalcitrance of the substrate^34^ and a corresponding low release of enzyme-inducing mono- or oligosaccharides. Genome analysis of *Thermomyces lanuginosus* revealed that it encodes few CAZy proteins (224) compared to other filamentous fungi (average 400)^35^. Glycoside hydrolases were significantly fewer in *Thermomyces lanuginosus* (94), whilst lignocellulosic fungi typically encode more than 200. Surprisingly, although *Thermomyces lanuginosus* is known as a xylanase superproducer, only a single β-1,4 xylanase was identified in its genome^35^. The zymography in our study indicated that strains of both *Thermomyces lanuginosus* and *Thermomyces dupontii* produce only one xylanase (xylanolytic band) when grown on untreated rice straw (Fig. 5). With its relatively small genome size, the percentage of CAZy proteins versus the total number of predicted proteins in *Thermomyces lanuginosus* is 4.38% and among the highest in fungi. Small genome size, and efficient gene regulation and enzyme transport are thought to be important factors in lowering the energy expenditure in fungi, which in turn is crucial in high-temperature environments^35^.

It is interesting to note that, the group of strains showing low CMCase activities exhibited a rather high maximum growth temperature (Fig. 4). Species like *Rhizomucor pusillus, Rhizomucor miehei, Rasamsonia emersonii, Thermomyces dupontii, Thermomyces lanuginosus* are known to involved in phase I of composting process, where readily accessible nutrients, microbial activity, and heat accumulation are at their maximum^21^.

Among the group of strains demonstrating high CMCase activity were species previously assigned to the genus *Myceliophthora* (*Crassicarpon thermophilum*, *Thermothelomyces heterothallica* and *Thermothelomyces thermophila*), species of *Thielavia* and the thermotolerant fungus *Aspergillus fumigatus* (Fig. 4). When cultivated on untreated rice straw, the strains produced high amounts of extracellular proteins. These strains also demonstrated relatively lower maximum growth temperatures, which indicates that they might be involved in the later stages of plant biomass degradation when more readily accessible nutrients have been exhausted and temperatures have dropped. *Thielavia* species and species previously under the genus *Myceliophthora* share such a high morphological resemblance during vegetative growth that several species of *Myceliophthora* were previously regarded as anamorphs of *Thielavia*^36^. In the present study, the two groups showed rather contrasting lignocellulolytic activity profiles. While *Thielavia* species exhibited both xylanase and CMCase activities, *Myceliophthora* species showed almost exclusively CMCase activity. The *Myceliophthora* genus was recently restructured based on ITS, translation elongation factor 1-α (TEF1) and RPB1 gene sequences. The species *Myceliophthora heterothallica* and *Myceliophthora thermophila* were placed under *Thermothelomyces* and *Myceliophthora fergusii* under *Crassicarpon*. Currently, *Myceliophthora* comprises only mesophilic species^37^. The thermotolerant *Aspergillus fumigatus* strains also demonstrated high CMCase and xylanase activities. This species is a human pathogen^38^ and its direct use in technical applications is therefore questionable. Among the thermophilic and thermotolerant fungi, *Aspergillus fumigatus* encodes the highest number of CAZymes^35^, and the genes encoding thermostable cellulases from this species might be worth pursuing^39^.

By using a very acidic medium at high temperature for isolation, we aimed to isolate fungi able to produce thermostable enzymes that can function under acidic conditions. Most of the isolates produced CMCases that exhibited higher activities at pH 3 than at pH 5 or pH 7, and were also thermostable. CMCase-exhibiting enzymes of 31 and 14 isolates retained more than 75% and 90% of their activity, respectively, after treatment at 70 °C for 20 min. Xylanases were generally less thermostable and showed lower activity under acidic conditions than CMCases (Fig. 4).

Heat treatment at 70 °C effectively denatured most of the xylanases, although xylanase produced by *Rasamsonia emersonii* LPH 067 was not affected, and xylanases produced by *R. emersonii* LPHT 227, *R. emersonii* LPHT 234, *Rhizopus microsporus* LPH 143 and *Mycothermus thermophilus* LPH 128 retained more than 75% of their initial activity. Similarly, 7 isolates (*Rh. microsporus* LPH 143, *Thielavia terrestris* FCH 9.4, *T. terrestris* LPHT 226, *Thielavia* sp. LPHT 232, *Thielavia* sp. LPHT 225, *Thielavia* sp. LPHT 235 and *Thielavia* sp. LPH 182) showed higher xylanase activities at pH 3 than at pH 5 and pH 7. Overall, the fungal secretomes produced by *Thielavia* spp. are of special interest because of their high enzymatic activity, thermostability, and the possibility of functioning under acidic conditions (Fig. 4).

In this study, rice straw was chosen as the substrate for lignocellulolytic enzyme production because it represents the most important lignocellulosic waste stream in Vietnam and other Southeast Asian countries^34^. Instead of pretreating rice straw and adding substrates facilitating optimal enzyme production, we chose minimum mineral medium with untreated rice straw as the medium for solid state fermentation. The ability of fungal isolates to colonize and degrade untreated rice straw provides insight into the natural process of plant biomass degradation by the fungi. Rice straw is especially recalcitrant to biodegradation, partly due to its high silica content^34^.

Fungal biodiversity in the tropics is high, but remains largely unstudied. Metagenomic analysis of soil samples collected around the globe has indicated that the highest fungal diversity is found in northwest Latin America, the southwest coast of India, on the Kalimantan island and in the triangle between Vietnam, Laos and China^40^. Characterisation of fungal diversity by isolation is often problematic since the real diversity may be masked by cosmopolitan species that occur at high frequency and cell density. By using a moderate selection pressure (elevated temperature, low pH and lignocellulosic substrate), we have here discovered numerous previously undocumented thermophilic fungi in the genus *Thielavia* which exhibited high genetic divergence. The selection medium was proven to be useful in finding fungi and enzymes with targeted properties. Studying secretomes produced by fungi on native substrates can provide new information on their roles in the natural processes of plant biomass degradation.

## Methods

### Isolation and cultivation of fungi

Various types of samples containing decaying plant residues (compost, grasses, rice straw, mushroom ground, wood, soil) were collected from different provinces in the northern part of Vietnam during the years 2012–2016. Fungi were isolated in a newly formulated medium containing untreated rice straw as the sole carbon source at pH 2.0 and 50 °C. For fungal isolation, samples were washed 3 times with sterile Czapek Dox mineral base ((NH_4_)_2_SO_4_ 1.0 g L^-1^; K_2_HPO_4_.3H_2_O 0.5 g L^−1^; KCl 0.25 g L^−1^; MgSO_4_.7H_2_O 0.25 g L^−1^, FeSO_4_.7H_2_O 5 mg L^−1^) with the pH adjusted to 2.0 using 1.0 M H_2_SO_4_. Washing was performed to adjust the pH of samples that may have buffering capacity, and to enhance the fungal diversity by removing the overload of aerial spores while retaining substrate mycelia. For the preparation of the isolation medium, rice straw (variety Japonica J02) was hammer-milled and passed through an 18-mesh sieve. It was then rinsed with acidified Czapek Dox mineral base, autoclaved at 121 °C for 15 min and spread on plastic Petri dishes to form a 5–8 mm semi-solid layer. The pre-washed samples were sprinkled over the rice-straw plates and incubated at 50 °C. To avoid evaporation, the incubation chamber was humidified by inserting large trays of water. After 7–10 days of incubation, the fungal colonies formed were transferred to potato dextrose agar (PDA) plates (HiMedia, India) and purified by hyphal tip culture. After cultivation in PDA slants at 50 °C, the isolates were maintained in a refrigerator at 2–8 °C. For long-term storage, a loop-full of fungal biomass was transferred to neutral glass tubes containing sterile washed sand. The contents were freeze-dried and the tubes were vacuum sealed. Fungal isolates can be stored in this way for several years.

To assess the growth profiles at different temperatures, fungi were inoculated on PDA plates and incubated at 20, 30, 35, 40, 45, 50, 55, and 60 °C for 7 days. The growth was assessed in terms of colony diameter. Growth at different pH was tested at 50 °C in liquid YM broth (glucose 10 g L^−1^, malt extract 3 g L^−1^, peptone 5 g L^−1^, yeast extract 3 g L^−1^) with the pH adjusted to 2.0, 2.5, 3.0, 3.5, 4.0, 4.5 and 5.0 using 1.0 M H_2_SO_4_. Growth was evaluated visually after 7 days of incubation.

### Solid state cultivation and enzyme extraction

For enzyme production, fungi were cultivated in a solid medium using untreated rice straw as the sole carbon source. Precultures were made by growing the fungi on PDA plates at 50 °C for 5 days. Surface mycelia and spores were scalped off and suspended in 4 mL of sterile 0.05% Tween 80 solution. One mililiter of the cell suspension was added to a 100 mL Erlenmeyer flask containing 5 g of milled rice straw and 10 mL of standard Czapek Dox mineral base (pH 5.0). The flasks were capped with SILICOSEN^®^ C-type lids (Shin-Etsu Polymer, Japan) and incubated at 50 °C for 7 days in a humidified chamber. The flask contents were mixed once per day by manual shaking. For enzyme extraction, 45 mL of 50 mM sodium citrate buffer at pH 5.0 was added to the flask. It was then shaken for 2 h at 30 °C in a rotary shaker at 160 rpm. The supernatant was obtained by centrifugation at 7 000 *g* for 10 min at 10 °C and stored at −30 °C until use.

### Hydrolytic activities

For the determination of xylanase or CMCase activity, 0.1 mL of crude culture filtrate was mixed with 0.2 mL of a 10 g L^−1^ xylan (from beechwood, Cas: 9014–63–5, Apollo Scientific) or 10 g L^−1^ CMC (Cas: 9004-32-4, Sigma) solution. After incubation at 50 °C for 20 min, 0.6 mL of DNS Reagent (water, 1416 mL; 3,5 dinitrosalicylic acid, 10.6 g; sodium hydroxide, 19.8 g; sodium potassium tartrate, 306 g; phenol, 7.6 mL; sodium metabisulphite, 8.3 g) was added and incubated at 100 °C for 5 min. The contents were cooled down, and 0.4 mL was mixed with 1.8 mL water, and the absorbance was measured at 540 nm^41^. Calibration curves were constructed using xylose and glucose for xylanase and CMCase activities, respectively. Britton-Robinson buffer (40 mM H_3_BO_3_, 40 mM H_3_PO_4_ and 40 mM CH_3_COOH) titrated to the desired pH with 0.2 M NaOH was used to determine the enzymatic activity at different pH (pH 3.0, 5.0, and 7.0). Crude culture filtrates were incubated at 70 °C for 20 min and the residual activity was measured to assess the thermostability.

Zymography was performed based on conventional denaturing SDS-PAGE^42,43^ using a 10% polyacrylamide gel containing 1% CMC or 1% xylan. After electrophoresis, to renature the protein fractions, the gel was rinsed twice in 2% Triton X-100 for 30 min at room temperature, and then twice with ice-cold 50 mM sodium citrate buffer, pH 5.0 for 15 min. In-gel hydrolysis was carried out by incubating the gel in 50 mM sodium citrate buffer, pH 5.0 at 50 °C for 1 h. The gel was then rinsed 4–5 times with distilled water and stained with 0.1% Congo Red solution at 50 °C for 1 h. De-staining was carried out with 1 M NaCl. Active CMCase or xylanase fractions appeared as transparent bands on the red background. Images were obtained using a digital camera (Nikon D7000) and converted into grayscale images. The images were overlaid as yellow (CMCase) and dark-blue (xylanase) channels in a new RGB image using Adobe Photoshop CS6 to display both the CMCase and xylanase activity.

### Identification of fungi

Fungi were identified by sequencing of the internally transcribed spacer (ITS) region. This region has been found to be among the markers with the highest probability of correct identification for a very broad group of fungi^23^. Since there is no definitive percentage of sequence similarity that precisely indicates conspecific taxa, we adopted the average weighted infraspecific ITS variability value of 2.51% for the fungi kingdom (1.96% for Ascomycota, and 3.24% for Zygomycota)^44^ as guidance in species assignment.

Fungi were grown on PDA for 3–5 days at 50 °C. For DNA extraction, a loopful of mycelium was transferred to a micro-tube containing 1 mL of 2 × SSC (15 mM sodium citrate, 150 mM NaCl, pH 7.0). The tubes were incubated at 99 °C for 10 min using a dry heating block (Grant-bio, England). Cells were collected by centrifugation at 10 000 *g* for 1 min. About 100 μL of glass beads (0.2–0.5 mm in diameter; Roth, Germany), 100 μL of phenol-chloroform (1:1, v/v) and 150 μL of water were added to the cell pellet. The cells were disrupted using a Mini-Beadbeater-8 (Biospec, USA) for 45 s. The tubes were centrifuged at 14 000 *g* at 4 °C for 10 min, and the upper layer was transferred to a new micro-tube. The DNA solution was further purified using a Silica Bead DNA Gel Extraction kit (Thermo Scientific) according to the manufacturer’s instruction.

PCR amplification and sequencing of the ITS region were done using one of the forward primers ITS1 (TCC GTA GGT GAA CCT GCG G)^45^, ITS5 (GGA AGT AAA AGT CGT AAC AAG G)^45^, ITS1F (CTT GGT CAT TTA GAG GAA GTA A)^46^ or SR6R (AAG WAA AAG TCG TAA CAA GG)^47^, and one of the reverse primers ITS4 (TCC TCC GCT TAT TGA TAT GC)^45^ or LR1 (GGT TGG TTT CTT TTC CT)^47^. The PCR cycle was as follows: 94 °C for 3 min; 30 cycles of 94 °C for 40 s, 52 °C for 40 s and 72 °C for 60 s; 72 °C for 10 min. The DNA sequencing service provided by the First BASE Laboratories Sdn Bhd (Selangor, Malaysia) was used. The sequences obtained have been deposited in GenBank with consecutive accession numbers from MH305194 to MH305299. The sequences were compared with the available databases using BLAST at the National Center for Biotechnology Information (https://blast.ncbi.nlm.nih.gov), the sequence-based identification tool at UNITE Community (https://unite.ut.ee) and the CBS-KNAW Pairwise sequence alignment identification tool (http://www.westerdijkinstitute.nl). For phylogenetic analysis, sequences were aligned with a fast Fourier transform algorithm using the online version of MAFFT^48^ (http://www.ebi.ac.uk/Tools/msa/mafft/) hosted at the European Bioinformatics Institute (Hinxton, Cambridgeshire, UK). Phylogenetic trees were constructed using MEGA7^49^. The graphical combination of enzymatic activities in the form of heat maps and a phylogenetic tree was performed using iTOL^50^.

## Supplementary information

Supplementary Dataset S1

Supplementary S2
