# Supplementary material for: Surveying of acid-tolerant thermophilic lignocellulolytic fungi in Vietnam reveals surprisingly high genetic diversity
Source: Sci Rep. 2019 Mar 6;9:3674. doi: 10.1038/s41598-019-40213-5 (PMC6403320; doi:10.1038/s41598-019-40213-5)
Supplement: Supplementary file 2 — Supplementary S2 [file 41598_2019_40213_MOESM2_ESM.pdf]

Surveying of acid-tolerant thermophilic lignocellulolytic fungi in Vietnam reveals surprisingly high genetic diversity

Vu Nguyen Thanh, Nguyen Thanh Thuy, Han Thi Thu Huong, Dinh Duc Hien, Dinh Thi My Hang, Dang Thi Kim Anh, Silvia Hüttner, Johan Larsbrink, and Lisbeth Olsson

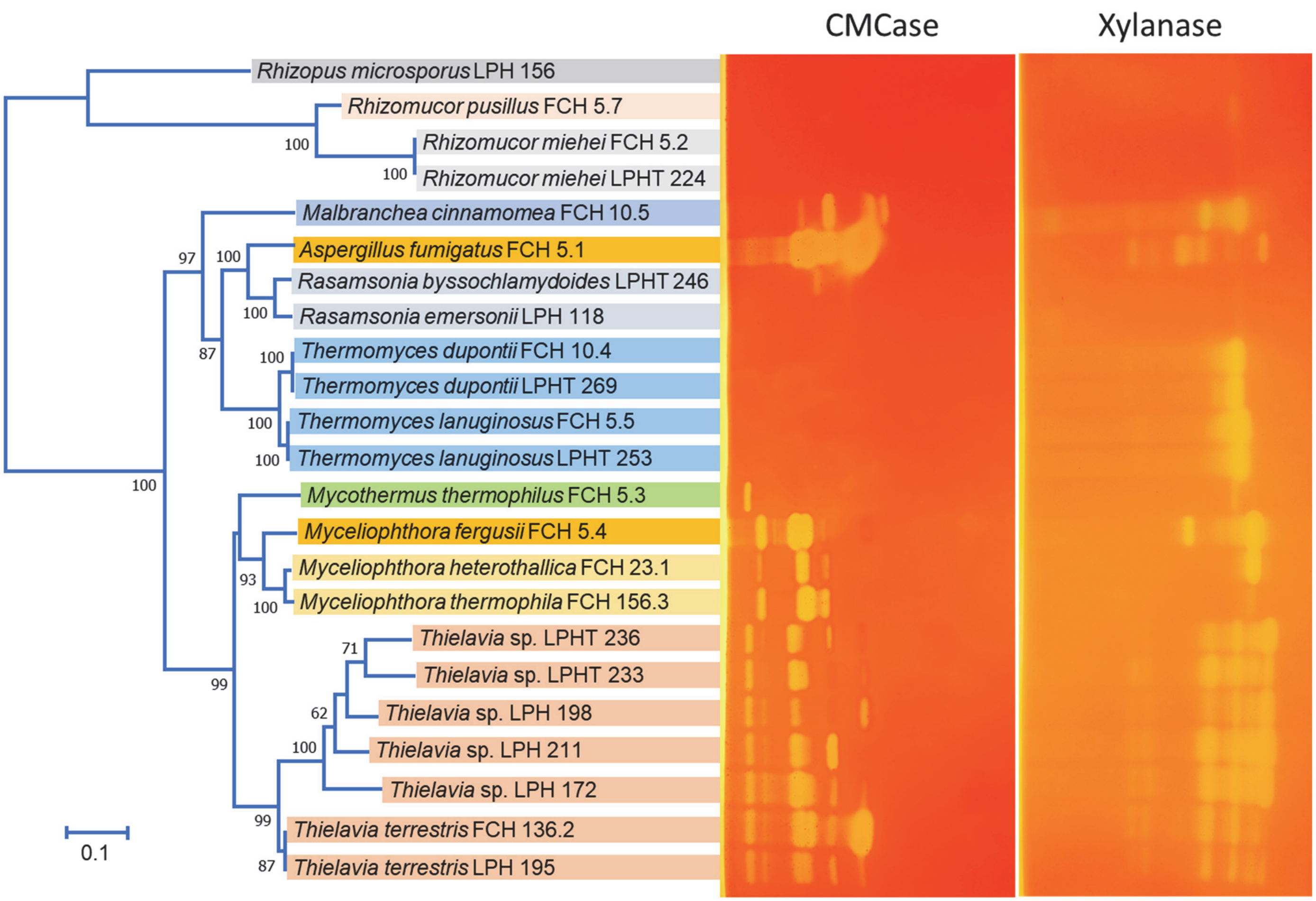

Supplementary S2. Zymograms of secretomes obtained from selected thermophilic and thermotolerant strains grown on rice straw as the sole carbon source. The neighbour-joining tree was constructed based on the ITS sequences using MEGA7. Bar, 10% sequence divergence.
